# Calcarea and the Proteins

**Published:** 1897-07

**Authors:** M. R. Leverson

**Affiliations:** New York


### CALCAREA AND THE PROTEINS.

NEW YORK, June 23d, 1897.

EDITOR THE HOMEOPATHIC PHYSICIAN.

In your editorial upon "Calcarea Carbonica," page 145, vol. XVII (May number), you give the usual "reason" for not prescribing Calc-carb. as a food, viz., that it must be combined with the proteins before it can be assimilated.

I am not able to accept this reasoning.

Gelatine sugar is able to combine with both acids and bases, which, when insoluble in water, become soluble by combining with the gelatine sugar of the blood or the albumen of the lymph (see Dr. Hensel's *Macrobiotic*, page 29).

Hence the lymph which contains gelatine sugar may select mineral substances from the food, transport them *in solution* to the parts where required, and there deposit them as the gelatine sugar combines with acids or the hydrocarbon becomes oxidized.

Hence while Calc-carb. should be given as a drug only when indicated by the law of *Similia*, I can see no objection and probably a positive gain in giving it as food in cases where it is evidently deficient—*e. g.*, rickets.

M. R. LEVERSON.

